# Synthesis, Characterization, and Investigation of Anti-Inflammatory and Cytotoxic Activities of Novel Thiourea Derivatives of Naproxen

**DOI:** 10.3390/pharmaceutics16010001

**Published:** 2023-12-19

**Authors:** Nikola Nedeljković, Miloš Nikolić, Petar Čanović, Milan Zarić, Radica Živković Zarić, Jelena Bošković, Marina Vesović, Jovana Bradić, Marijana Anđić, Aleksandar Kočović, Marina Nikolić, Vladimir Jakovljević, Zorica Vujić, Vladimir Dobričić

**Affiliations:** 1 Department of Pharmacy, Faculty of Medical Sciences, University of Kragujevac, Svetozara Markovića 69, 34000 Kragujevac, Serbia; nikola.nedeljkovic@medf.kg.ac.rs (N.N.); marina.mijajlovic@medf.kg.ac.rs (M.V.); jovanabradic@medf.kg.ac.rs (J.B.); marijana.andjic@medf.kg.ac.rs (M.A.); salek@medf.kg.ac.rs (A.K.); 2 Department of Biochemistry, Faculty of Medical Sciences, University of Kragujevac, Svetozara Markovića 69, 34000 Kragujevac, Serbia; zaricmilan@medf.kg.ac.rs; 3 Department of Pharmacology and Toxicology, Faculty of Medical Sciences, University of Kragujevac, Svetozara Markovića 69, 34000 Kragujevac, Serbia; iasst_a8@medf.kg.ac.rs; 4 Department of Pharmaceutical Chemistry, Faculty of Pharmacy, University of Belgrade, Vojvode Stepe 450, 11221 Belgrade, Serbia; jelena.boskovic@pharmacy.bg.ac.rs (J.B.); zorica.vujic@pharmacy.bg.ac.rs (Z.V.); vladimir.dobricic@pharmacy.bg.ac.rs (V.D.); 5 Center of Excellence for Redox Balance Research in Cardiovascular and Metabolic Disorders, Svetozara Markovića 69, 34000 Kragujevac, Serbia; marina.nikolic@medf.kg.ac.rs (M.N.); vladimir.jakovljevic@medf.kg.ac.rs (V.J.); 6 Department of Physiology, Faculty of Medical Sciences, University of Kragujevac, Svetozara Markovića 69, 34000 Kragujevac, Serbia; 7 Department of Human Pathology, First Moscow State Medical University IM Sechenov, 119435 Moscow, Russia

**Keywords:** naproxen, thiourea, anti-inflammatory activity, paw edema, cytotoxic activity, MTT, apoptosis, COX-2, 5-LOX

## Abstract

The objective of this study was to synthesize seven novel thiourea derivatives of naproxen (**8**–**14**), examine the anti-inflammatory activity of the newly synthesized compounds, investigate the cytotoxic potential of both sets of synthesized compounds (**1**–**7** and **8**–**14**), and select the most promising anti-inflammatory and antitumor drug candidates. The results of the in vivo anti-inflammatory study clearly showed that compounds **8** and **9** were capable of decreasing paw edema, as evident from a high percentage of inhibition (44.83% and 49.29%, respectively). In addition, the results of in vitro enzyme inhibition assays demonstrated that neither of the newly synthesized compounds reached 50% inhibition of 5-LOX at concentrations lower than 100 µM. In terms of antitumor potential, derivatives **3** and **8** exhibited strong cytotoxic effects on the HeLa cell line, suggesting the involvement of the extrinsic pathway of apoptosis. According to the overall results obtained for both sets of synthesized molecules, derivatives **4** and **8** can be underlined as molecules with the strongest anti-inflammatory activity, while derivatives **3** and **8** are the most promising cytotoxic agents.

## 1. Introduction

Inflammation represents the physiological response of the human body to various types of injury, including infection, physical and ischemic trauma, as well as exposure to different toxins. This protective reaction of the body triggers cellular changes in the inflamed tissue, resulting in the repair of the injury and consequent cell proliferation at the site of inflammation [1,2]. However, if the origin of inflammation is maintained, acute inflammation is converted into the pathophysiological chronic inflammation [3]. It is well known that chronic inflammation and its mediators have been associated with the crucial steps involved in tumor development, invasion, and survival [4,5].

The fundamental components of cancer-related inflammation comprise the infiltration of white blood cells, various cytokines and chemokines, macrophages, acceleration of the cell cycle and cell proliferation, avoidance of apoptosis, and pathological angiogenesis stimulation [6,7]. In line with that, there is increased evidence that certain non-steroidal anti-inflammatory drugs (NSAIDs), such as aspirin, reduce mortality and the risk of various cancers [8,9,10,11]. It is well established that certain NSAIDs have beneficial effects in the chemoprevention of colorectal cancer due to their inhibition activity against cyclooxygenase-2 (COX-2) isoform, which is overexpressed in this tumor disease. Through COX-2 inhibition, the synthesis of the prostaglandin E_2_ is suppressed, which results in the reduction of cell proliferation and in the increase of apoptosis [12]. In addition, COX-1 inhibition has been proposed as another possible mechanism of cancer prevention through the avoidance of hematogenous tumor dissemination [13]. Although healthy tissues do not express 5-lipoxygenase (5-LOX) enzyme (with the exception of leukocytes), overexpression of this enzyme and other mediators involved in leukotriene biosynthesis has been observed in several solid cancers [14,15,16,17,18]. Moreover, it was shown that elevated expression of 5-LOX corresponds with tumor size, metastatic potential, decreased efficacy of cytostatic therapy, and poor prognosis [19]. In accordance with the above facts, dual COX-2 and 5-LOX inhibitors may be a useful therapeutic alternative compared to conventional cytostatic therapy in the combat against the progression of numerous tumor diseases [20].

NSAIDs are a large class of drugs with wide structural and functional diversity, mainly used for the treatment of patients suffering from pain and inflammatory conditions. In general, the structure of NSAIDs consists of an acidic moiety attached to a planar aromatic functional group [21]. In the modern synthesis of NSAID derivatives, the focus is on masking the carboxyl functional group because the results of studies have shown that this acidic group is associated with the toxic effect [22,23]. The coupling of this pharmacophore with sulfonamides increases the COX-2 selectivity of obtained compounds and decreases their ulcerogenic potential [24].

Naproxen is a propionic acid derivative that unselectively inhibits the two major cyclooxygenases (COX-1 and COX-2) and can reduce tumor growth in tumor-bearing rats [25]. Previously published studies have shown that urea and propanamide derivatives of naproxen possess significant inhibitory potential against the colon cancer cell line HCT-116 [26,27]. On the other hand, ethanamide derivatives of naproxen demonstrated dose-dependent apoptosis of these cells by inhibiting COX-1 and COX-2 enzymes [28]. The previously mentioned propanamide derivatives inhibited the growth of the breast cancer cell line MDA-MB-231 through apoptosis compared to their parent molecule naproxen [26]. Nitrophenyl and hydroxyphenyl oxadiazole derivatives of naproxen showed highly potent cytotoxic activity against MCF-7, MDA-MB-231, HeLa, and HCT-116, comparable to the reference drugs. For example, doxorubicin, afatinib, and celecoxib yielded IC_50_ values of 3.18–26.79, 6.20–11.40, and 22.79–42.74 μM, respectively [29].

Thiourea derivatives are promising classes of anticancer and anti-inflammatory agents due to their inhibitory potential against tyrosine kinases (PTKs) [30], human sirtuin proteins type 1 and 2 (SIRT1 and SIRT2) [31], as well as cyclooxygenases and lipoxygenase [32]. Namely, the most active anticancer drugs belong to the derivatives of thiourea, urea, and benzothiazole [33]. Thioureas in combination with benzothiazoles showed significant anti-cancer activity against Hela and MCF-7 cells by inhibiting DNA topoisomerase [34]. In terms of anti-inflammatory activity, thiourea derivatives of naproxen with 4-chloroaniline [35], aminopyridines [36], and amino acids [27] exhibited higher anti-inflammatory activity in reducing rat paw edema concerning the standard drug, naproxen.

In our previously published paper [37], the synthesis of seven thiourea derivatives of naproxen (**1**–**7**) was reported, as well as the investigation of their anti-inflammatory activity using in vivo, in vitro, and in silico approaches. Our strategy for the synthesis of these derivatives was based on the conversion of naproxen’s carboxyl group to the thiourea moiety using lipophilic and sterically bulky aromatic amines and esters of aromatic amino acids. The aim of this research was to synthesize seven novel thiourea derivatives of naproxen (**8**–**14**), examine the anti-inflammatory activity of newly synthesized compounds, investigate the antitumor potential of both sets of synthesized compounds (**1**–**7** and **8**–**14**), and select the best anti-inflammatory and antitumor drug candidates. In order to get deeper insight into the anti-inflammatory effects of naproxen derivatives, in vitro assays of COX-2 and 5-LOX inhibition were carried out, while apoptotic mechanisms and potential effects on the cell cycle of HeLa cells were also studied in detail.

## 2. Materials and Methods

### 2.1. Chemicals and Instruments

Potassium thiocyanate, phenylalanine methyl ester hydrochloride, dichloromethane, and N,N-dimethylformamide were purchased from Acros Organics (Geel, Belgium), while S-naproxen, oxalyl chloride, aniline, *p*-toluidine, *o*-toluidine, *o*-anisidine, *o*-fluoroaniline, tryptophan methyl ester hydrochloride, acetone, and carrageenan were purchased from Sigma-Aldrich (Steinheim, Germany). Methanol and chloroform were obtained from JT Baker (Loughborough, UK), whereas silica gel used for preparative thin-layer chromatography (TLC) was obtained from Merck (Darmstadt, Germany). Structural characterization of thiourea derivatives of naproxen was performed by measuring melting points, as well as using IR, NMR, HRMS, and MS/MS spectroscopic methods. A Boetius PHMK 05 apparatus (Radebeul, Germany) was used for determining melting points. The ATR-FTIR spectrometer Nicolet iS10 (Thermo Scientific, Madison, WI, USA) was used to record IR spectra, while NMR Bruker Avance III spectrometer (Bruker Biospin GmbH, Rheinstetten, Germany) was used for recording ^1^H NMR (400 MHz) and ^13^C NMR (100 MHz) spectra. Determination of exact masses was performed using an LTQ Orbitrap XL Mass Spectrometer (Thermo Fisher Scientific, Bremen, Germany), while a TSQ Quantum Access MAX triple quadrupole mass spectrometer (Thermo Fisher Scientific, San Jose, CA, USA), equipped with a heated electrospray ionization source (HESI), was used for MS/MS analyses.

### 2.2. Synthetic Procedures

The new series of naproxen thiourea derivatives (**8**–**14**) was synthetized based on the previously described synthetic route [37]. Naproxenoyl-chloride was obtained in the reaction of *S*-naproxen, dissolved in dichloromethane, and oxalyl-chloride with the addition of a catalytic amount of DMF at room temperature. Compounds **8**–**12** were synthesized by adding 0.5 mmol of aromatic amine (aniline for compound **8**, *p*-toluidine for compound **9**, *o*-toluidine for compound **10**, *o*-anisidine for compound **11**, and *o*-fluoroaniline for compound **12**) dissolved in anhydrous acetone to the reaction mixture obtained by mixing acetone solutions of naproxenoyl-chloride and potassium-thiocyanate. Amino acid esters derivatives (**13** and **14**) were obtained by adding 0.5 mmol acetone solution of amino acid ester hydrochloride (phenylalanine methyl ester for compound **13** and tryptophan methyl ester for compound **14**) and trimethylamine (0.05 mmol) to the mixture of naproxenoyl-chloride and potassium-thiocyanate prepared in acetone. The reaction mixture was refluxed using acetone for three hours, afterwards evaporating to dryness at room temperature was carried out. After dissolving in chloroform, purification of reaction mixtures was conducted using preparative thin-layer chromatography (TLC), whereby chloroform (compounds **8**–**10**, **12**), dichloromethane/methanol 99:1, *v*/*v* (compound **11**), and chloroform/methanol 99:1, *v*/*v* (compounds **13** and **14**) were used as mobile phases.

*(S)-2-(6-methoxynaphthalen-2-yl)-N-(phenylcarbamothioyl)propanamide* (**8**). Yield: 28%. Light-yellow crystalline solid. Melting point: 137–139 °C. IR (ATR) ν_max_ (cm^−1^): 1149.16, 1688.36, 3031.36, and 3169.36. **^1^H NMR (400 MHz, DMSO-d_6_) δ ppm** 1.50 (3H, d, *J* = 6.8, C**H_3_**_Nap_), 3.86 (3H, s, OC**H_3_**_Nap_), 4.22 (1H, q, *J* = 6.8, C**H**_Nap_), 7.15–7.84 (11H, m, Ar**H**), 11.67 (1H, s, N**H**CS), and 12.43 (1H, s, CSN**H**) (Figure S1a). **^13^C NMR (100 MHz, DMSO-d_6_) δ ppm** 18.52 (**C**H_3Nap_), 45.45 (**C**H_Nap_), 55.65 (O**C**H_3Nap_), 106.20–157.76 (aromatic carbons), 176.65 (**C**ONH), and 179.46 (NH**C**S) (Figure S1b). *m*/*z* = 364.8 [M + H]^+^, 364.06, 229.90, 184.84, 169.83, 154.05, and 152.88. MS [M + Na]^+^ calculated for C_21_H_20_N_2_O_2_S = 387.11377; observed = 387.11342.
*(S)-2-(6-methoxynaphthalen-2-yl)-N-(p-tolylcarbamothioyl)propanamide* (**9**). Yield: 26%. Light-yellow crystalline solid. Melting point: 123–124 °C. IR (ATR) ν_max_ (cm^−1^): 1154.72, 1682.19, 3023.16, and 3176.53. **^1^H NMR (400 MHz, DMSO-d_6_) δ ppm** 1.50 (3H, d, *J* = 7.2, C**H_3_**_Nap_), 2.29 (3H, s, C**H_3_**), 3.87 (3H, s, OC**H_3_**_Nap_), 4.22 (1H, q, *J* = 6.8, C**H**_Nap_), 7.16–7.85 (10H, m, Ar**H**), 11.64 (1H, s, N**H**CS), and 12.37 (1H, s, CSN**H**) (Figure S2a). **^13^C NMR (100 MHz, DMSO-d_6_) δ ppm** 17.45 (**C**H_3Nap_), 19.96 (**C**H_3_), 44.29 (**C**H_Nap_), 54.59 (O**C**H_3Nap_), 105.14–156.69 (aromatic carbons), 175.56 (**C**ONH), and 178.27 (NH**C**S) (Figure S2b). *m*/*z* = 379.1 [M + H]^+^, 230.07, 185.06, 169.95, 167.10, 149.98, and 108.19. MS [M + Na]^+^ calculated for C_22_H_22_N_2_O_2_S = 401.12942; observed = 401.12959.
*(S)-2-(6-methoxynaphthalen-2-yl)-N-(o-tolylcarbamothioyl)propanamide* (**10**). Yield: 42%. Yellow crystalline solid. Melting point: 110–110.5 °C. IR (ATR) ν_max_ (cm^−1^): 1157.26, 1686.27, 3025.90, and 3177.30. **^1^H NMR (400 MHz, DMSO-d_6_) δ ppm** 1.50 (3H, d, *J* = 6.8, C**H_3_**_Nap_), 2.17 (3H, s, CH_3_), 3.86 (3H, s, OC**H_3_**_Nap_), 4.22 (1H, q, *J* = 6.8, C**H**_Nap_), 7.15–7.84 (10H, m, Ar**H**), 11.68 (1H, s, N**H**CS), and 12.08 (1H, s, CSN**H**) (Figure S3a). **^13^C NMR (100 MHz, DMSO-d_6_) δ ppm** 18.02 (**C**H_3Nap_), 18.61 (**C**H_3_), 45.43 (**C**H_Nap_), 55.66 (O**C**H_3Nap_), 106.22–157.77 (aromatic carbons), 176.58 (**C**ONH), and 180.30 (NH**C**S) (Figure S3b). *m*/*z* = 379.2 [M + H]^+^, 230.13, 185.12, 170.08, 167.09, 153.11, and 150.08. MS [M + Na]^+^ calculated for C_22_H_22_N_2_O_2_S = 401.12942; observed = 401.12956.
*(S)-2-(6-methoxynaphthalen-2-yl)-N-((2-methoxyphenyl)carbamothioyl)propanamide* (**11**). Yield: 38%. Light-yellow crystalline solid. Melting point: 159–160 °C. IR (ATR) ν_max_ (cm^−1^): 1156.80, 1684.26, 2976.29, and 3208.73. **^1^H NMR (400 MHz, DMSO-d_6_) δ ppm** 1.50 (3H, d, *J* = 6.8, C**H_3_**_Nap_), 3.84 (3H, s, OC**H_3_**), 3.86 (3H, s, OC**H_3_**_Nap_), 4.22 (1H, q, *J* = 6.8, C**H**_Nap_), 6.94–8.49 (10H, m, Ar**H**), 11.63 (1H, s, N**H**CS), and 12.71 (1H, s, CSN**H**) (Figure S4a). **^13^C NMR (100 MHz, DMSO-d_6_) δ ppm** 18.44 (**C**H_3Nap_), 45.37 (**C**H_Nap_), 55.65 (O**C**H_3Nap_), 56.47 (O**C**H_3_), 106.18–157.75 (aromatic carbons), 176.47 (**C**ONH), and 178.24 (NH**C**S) (Figure S4b). *m*/*z* = 395.3 [M + H]^+^, 230.18, 185.19, 183.18, 170.20, 153.22, and 124.24. MS [M + Na]^+^ calculated for C_22_H_22_N_2_O_3_S = 417.12433; observed = 417.12460.
*(S)-N-((2-fluorophenyl)carbamothioyl)-2-(6-methoxynaphthalen-2-yl)propanamide* (**12**). Yield: 33%. White crystalline solid. Melting point: 146–147 °C. IR (ATR) ν_max_ (cm^−1^): 1144.87, 1685.51, 2937.68, and 3183.02. **^1^H NMR (400 MHz, DMSO-d_6_) δ ppm** 1.52 (3H, d, *J* = 6.8, C**H_3_**_Nap_), 3.87 (3H, s, OC**H_3_**_Nap_), 4.23 (1H, q, *J* = 7.2, C**H**_Nap_), 7.16–8.00 (10H, m, Ar**H**), 11.85 (1H, s, N**H**CS), and 12.34 (1H, s, CSN**H**) (Figure S5a). **^13^C NMR (100 MHz, DMSO-d_6_) δ ppm** 17.45 (**C**H_3Nap_), 44.37 (**C**H_Nap_), 54.60 (O**C**H_3Nap_), 105.15–156.72 (aromatic carbons), 175.66 (**C**ONH), and 179.43 (NH**C**S) (Figure S5b). *m*/*z* = 383.1 [M + H]^+^, 230.14, 185.10, 171.07, 169.13, 153.11, and 152.06. MS [M + Na]^+^ calculated for C_21_H_19_FN_2_O_2_S = 405.10435; observed = 405.10465.
*Methyl (((S)-2-(6-methoxynaphthalen-2-yl)propanoyl)carbamothioyl)phenylalaninate* (**13**). Yield: 30%. White crystalline solid. Melting point: 58–60 °C. IR (ATR) ν_max_ (cm^−1^): 1173.47, 1693.18, 1742.27, 2933.81, and 3183.77. **^1^H NMR (400 MHz, DMSO-d_6_) δ ppm** 1.43 (3H, d, *J* = 6.8, C**H_3_**_Nap_), 3.15 (2H, dd, *J* = 5.6, *J* = 10, C**H_2_**_-Phe_), 3.64 (3H, s, R-C(=O)OC**H_3_**), 3.86 (3H, s, OC**H_3_**_Nap_), 4.10 (1H, q, *J* = 6.4, C**H**_Nap_), 5.05 (1H, q, *J* = 6.4, C**H**_-Phe_), 7.05–7.82 (11H, m, Ar**H**), 10.97 (1H, s, N**H**CS), and 11.59 (1H, s, CSN**H**) (Figure S6a). **^13^C NMR (100 MHz, DMSO-d_6_) δ ppm** 18.61 (**C**H_3Nap_), 36.63 (**C**H_2-Phe_), 45.39 (**C**H_Nap_), 52.73 (R-C(=O)O**C**H_3_), 55.66 (O**C**H_3Nap_), 58.95 (**C**H_-Phe_), 106.21–157.75 (aromatic carbons), 170.79 (R-**C**(=O)OCH_2_CH_3_), 176.50 (**C**ONH), and 180.82 (NH**C**S) (Figure S6b). *m*/*z* = 451.1 [M + H]^+^, 419.11, 391.28, 185.06, 180.16, 169.90, and 153.19. MS [M + Na]^+^ calculated for C_25_H_26_N_2_O_4_S = 473.15055; observed = 473.15096.
*Methyl (((S)-2-(6-methoxynaphthalen-2-yl)propanoyl)carbamothioyl)tryptophanate* (**14**). Yield: 32%. White crystalline solid. Melting point: 91–93 °C. IR (ATR) ν_max_ (cm^−1^): 1154.16, 1683.78, 1740.19, 3017.40, and 3190.00. **^1^H NMR (400 MHz, DMSO-d_6_) δ ppm** 1.41 (3H, d, *J* = 6.8, C**H_3_**_Nap_), 3.16 (2H, dd, *J* = 4.0, *J* = 12, C**H_2_**_-Trp_), 3.63 (3H, s, R-C(=O)OC**H_3_**), 3.88 (3H, s, OC**H_3_**_Nap_), 4.12 (1H, q, *J* = 6.8, C**H**_Nap_), 5.10 (1H, q, *J* = 6, C**H**_-Trp_), 6.69–7.82 (11H, m, Ar**H**), 10.94 (1H, s, N**H**CS), 11.00 (1H, s, N**H**_-Trp_), and 11.58 (1H, s, CSN**H**) (Figure S7a). **^13^C NMR (100 MHz, DMSO-d_6_) δ ppm** 17.54 (**C**H_3Nap_), 25.91 (**C**H_2-Trp_), 44.28 (**C**H_Nap_), 51.64 (R-C(=O)O**C**H_3_), 54.61 (O**C**H_3Nap_), 57.82 (**C**H_-Trp_), 105.14–156.68 (aromatic carbons), 170.12 (R-**C**(=O)OCH_3_), 175.33 (**C**ONH), and 179.65 (NH**C**S) (Figure S7b). *m*/*z* = 490.2 [M + H]^+^, 329.12, 261.06, 202.09, 185.10, 170.09, and 160.11. MS [M + Na]^+^ calculated for C_27_H_27_N_3_O_4_S = 512.16145; observed = 512.16195.

### 2.3. In Vivo Studies in Wistar Albino Rats: Toxicity and Anti-Inflammatory Potential 

In vivo examinations of safety evaluation and anti-inflammatory potential of novel compounds **8**–**14** were performed at the Center of Excellence for Redox Balance Research in Cardiovascular and Metabolic Disorders, University of Kragujevac, Serbia. Animals used in the current research were obtained from the Military Medical Academy, Belgrade, Serbia. They were housed at controlled conditions: temperature of 22 ± 2 °C with 12 h of automatic illumination daily. Experimental animals consumed commercial rat food (20% protein rat food; Veterinary Institute Subotica, Serbia) and water ad libitum. The experimental research was performed in accordance with the regulations of the Faculty’s Ethical committee for the welfare of laboratory animals (protocol code 01-10742, approval date: 14 October 2021) and principles of the Good laboratory practice and European Council Directive (86/609/EEC).

#### 2.3.1. Acute Oral Toxicity Assessment

The acute oral toxicity was assessed in healthy male rats (n = 24, three rats per group, body weight 180–200 g) according to a previously established protocol and the OECD Guidelines No. 423 [37,38]. 

Animals were randomly assigned to the following groups: control group—rats that received single dose of 1% DMSO and experimental groups—rats that received compounds **8**–**14** per os in the single dose of 10 mg/kg dissolved in 1% DMSO. All rats were fasted prior to administration of compounds and during 3 h following the treatment. 

Afterwards, rats were kept in single cages and observed once daily during 14 days for clinical signs of toxicity. All behavioral changes and any signs of toxicity were recorded (fur and skin, urination (color), feces consistency, eyes, salivation, any alterations in behavior pattern). Additionally, food and water intake and the body weight were measured daily over 14 days. After accomplishing the monitoring period, rats were sacrificed, and organ weight (liver, kidney, stomach, and heart) was recorded [39,40]. Relative organ weight was calculated as follows:

(absolute organ weight × 100%)/body weight of rat on the day of sacrifice(1)

#### 2.3.2. Assessment of In Vivo Anti-Inflammatory Potential 

The Carrageenan-induced rat paw edema model was used to assess the anti-inflammatory potential of novel compounds **8**–**14**. Induction of inflammation was performed in all animals by intraplantar injection of 1 mL 0.5% carrageenan saline into the left hind paw. 

Animals (n = 200) were randomly divided into the following groups: control groups, that received either 1% DMSO or naproxen, and experimental groups, that received the novel compounds **8**–**14**.

Control groups included DMSO and naproxen groups. Rats belonging to the DMSO group received 1% DMSO in the same volume as the compounds **8**–**14**, 60 min before carrageenan injection. Moreover, naproxen groups included rats that were subjected to naproxen treatment in three doses: 2.5 mg/kg, 5 mg/kg, and 10 mg/kg. Naproxen was applied in the same manner as 1% DMSO.

Experimental groups included rats that were treated with compounds **8**–**14** and they were divided into three subgroups depending on the applied dose: 2.5 mg/kg, 5 mg/kg, and 10 mg/kg. The examined compounds were dissolved in 1% DMSO prior to treatment and applied to rats 60 min before inducing inflammation, i.e., before carrageenan injection. Doses of naproxen and investigated compounds were defined based on our previously published research [37].

In order to quantify the anti-inflammatory effects of the tested compounds, left paw thickness was measured at specific time points: prior to inflammation induction (moment 0) and 1, 2, 3, and 4 h (moments 1, 2, 3, and 4) following carrageenan injection. Tissue thickness was measured in the middle of the rat paw using a Digital Vernier caliper (Aerospace, China). The percentage of inhibition of paw edema after treatment with different agents was calculated according to the formula, Equation (2):

% Inhibition = 100 × [1 − (Yt/Yc)](2)

where Yt = average increase in paw thickness in the treated group of rats between two measurement moments, and Yc = average increase in paw thickness in the untreated group of rats between two measurement moments [37,41].

### 2.4. Investigation of COX-2 and 5-LOX Inhibitory Activity

The COX-2 and 5-LOX inhibitory potential of the tested compounds was evaluated using a fluorometric COX-2 and 5-LOX inhibitor screening kit (Abcam, Cambridge, UK). These tests are based on determining the presence of prostaglandin G2 (a product of COX enzyme activity) or an intermediate generated by the enzyme 5-LOX, using fluorometric detection. The mentioned experiments were performed according to the manufacturer’s instructions [42,43].

For the determination of COX-2 inhibitory potential of tested compounds, stock solutions (5 mM in DMSO) were diluted with the same solvent (test solutions). The test solutions were further diluted with COX assay buffer by mixing 2 µL of the corresponding test solution and 8 µL of COX assay buffer. For the preparation of inhibitory control (IC), celecoxib (2 µL) and COX assay buffer (8 µL) were mixed. Solvent control (SC) was prepared by mixing DMSO (2 µL) and the COX assay buffer (8 µL), enzyme control (EC) was 10 µL of COX assay buffer, while the samples (S) were 10 µL of diluted test solutions. IC, SC, EC, and S were transferred into corresponding wells. For the determination of 5-LOX inhibitory potential of tested compounds, stock solutions of the same concentration (5 mM) were prepared in DMSO. These solutions were further diluted with the same solvent to obtain the test solutions, which were added into the appropriate wells (2 µL). EC, SC, and IC were prepared by adding 2 µL of the assay buffer, DMSO, and zileuton into corresponding wells, respectively.

The fluorescence of the samples was measured kinetically at 25 °C (COX-2: Ex/Em = 535/587 nm, during 10 min; 5-LOX: Ek/Em = 500/536 nm during 20 min) using a Synergi LX multi-mode microplate reader (BioTek, Shoreline, WA, USA).

### 2.5. In Vitro Cytotoxicity Studies

#### 2.5.1. Cell Cultures

Three cancer cell lines (MDA-MB-231, HCT116 and HeLa; i.e., human breast cancer cells, human colorectal cancer cells, and human cervical cancer cells, respectively)*,* and one non-malignant fibroblast MRC-5 cell line (control) were used in this study. The American Type Cell Collection (ATCC, Manassas, VA, USA) provided all examined cell lines. The cells were grown in a complete medium made up of high-glucose DMEM media supplemented with 10% fetal bovine serum and 200 mM L-glutamine (both from Sigma-Aldrich, USA). The cells were cultured in 25 cc flasks (Thermo Fischer Scientific, Waltham, MA, USA) at 37 °C with 100% humidity in a 5% CO_2_ atmosphere.

#### 2.5.2. MTT Assay

The MTT test was used to assess the cytotoxicity of compounds **1**–**14** [44]. The cytotoxicity of naproxen was also investigated due to its clinical use and structural similarity. Throughout the exponential development period, cells were taken from culture flasks and counted, and 5 × 10^3^ cells/well were planted into 96-well culture plates. Following that, the cell lines were treated with varying doses of the investigated chemicals (**1**–**14**), naproxen (0.3–100 M), and fresh complete media as a control. All evaluated cells were incubated for 24, 48, and 72 h at 37 °C in an environment containing 5% CO_2_ and 100% humidity. Following incubation, each well’s culture media was taken out, each well received an addition of MTT solution at a final concentration of 0.5 mg/mL, and the cells were left to incubate for an additional four hours. The MTT solution was then withdrawn carefully, and the formazan crystals were dissolved in DMSO. After shaking the plates for 10 min, absorbance at 595 nm was measured using a multiplate reader (Zenyth 3100, Anthos Labtec Instruments, Salzburg, Austria). All concentrations of the tested compounds were performed in three separate wells of the microtiter plates and in three separate experiments. The results were reported as a ratio to the control value, untreated cells. IC_50_ values were also determined using Microsoft Office Excel 2021. The percentage of viable cells was estimated by dividing the readout absorbance value in the wells containing treated cells by the average absorbance value measured in the wells containing untreated cells, and multiplying the result by 100 [44].

% of the viable cells = ((absorbance of treated cell-absorbance of blank)/(absorbance of untreated cell-absorbance of blank)) × 100(3)

#### 2.5.3. Annexin V/7AAD Assay

The type of cell death induced by compounds **3** and **8** was determined by the annexin V–fluorescein isothiocyanate (FITC)/propidium iodide (PI) Apoptosis Kit (BD Biosciences, Franklin Lakes, NJ, USA) [44]. HeLa cells were incubated with the previously calculated IC_50_ values of these two compounds or with media alone for 24 h at 37 °C in an atmosphere of 5% CO_2_ and at absolute humidity. Furthermore, tested HeLa cells were trypsinized, washed in PBS, centrifuged, and resuspended in 100 μL of ice-cold binding buffer. The tested cells were then stained with both 10 μL of Annexin V-FITC and 20 μL of 7-AAD, incubated for 15 min in the dark at room temperature, and 400 μL of binding buffer was added to each tube. The obtained samples were measured with a flow cytometer Cytomics FC500 (Beckman Coulter, Brea, CA, USA). In addition, the obtained data were analyzed using FlowJo V10 Software. Measurements were presented as the representative density plots of Annexin V-FITC and PI staining and the average values of three independent experiments [44].

#### 2.5.4. Cell Cycle Analysis

The next step in this study was to evaluate whether the rate of division of HeLa tumor cells slows down under the influence of tested compounds. To examine the potential effect of compounds **3** and **8** on the cell cycle of HeLa cells, the cell line was incubated and treated with previously calculated IC_50_ values. The control population of untreated cells was also harvested and washed in the above-mentioned manner. Furthermore, HeLa cells were harvested, washed with PBS, and fixed with 70% ethanol at +4 °C. Cells were agglomerated and finally resuspended in 1 mL PBS containing RNAse A (500 μg/mL). After 30 min of incubation, cells were stained with 5 μL PI (10 mg/mL PBS). After 15 min of incubation in the dark, the samples were examined using a Beckman Coulter FC500 flow cytometer. FlowJo Software was used to analyze the cell cycle distribution, and the results were displayed as representative histograms and average values of three independent experiments [44].

#### 2.5.5. Assessment of Apoptosis

The aim of this study was to determine the expression of proteins that play a key role in apoptosis. The level of expression of the proapoptotic protein Bax, antiapoptotic protein Bcl-2, and the percentage of cells containing active caspase-3 was evaluated. Tested HeLa cells were incubated for 24 h and treated with IC_50_ concentration of the tested compounds or in a complete cell culture medium (control). Additionally, HeLa cells were fixed, permeabilized, and rinsed with ice-cold PBS (Fixation and Permeabilization Kit, eBioscience, San Diego, CA, USA). The cells were treated for 15 min at room temperature with a 1:1000 Bcl-2 fluorescein isothiocyanate (FITC) primary antibody (mhbcl01, Life Technologies, Carlsbad, CA, USA) to stain them. For further labeling, permeabilized HeLa cells were incubated with primary antibodies for active-Bax (N20, sc-493; Santa Cruz Biotech Inc., Dallas, TX, USA) and cleaved caspase-3 (#9661, Cell signaling Technology, Danvers, MA, USA) at a ratio of 1:1000 for 30 min. Furthermore, HeLa cells were treated with PBS before being incubated for 30 min with the 1:2000 secondary goat anti-rabbit IgG-FITC antibody (Ab6717-1, Abcam). Following that, the cells underwent a PBS wash and flow cytometry analysis. With the FC500 (Beckman Coulter), fluorescence of at least 15,000 events/samples had been measured. Negative control antibodies that matched the isotype were used to normalize the fluorescence intensity. The degree of expression of these proteins was indicated by the mean fluorescence intensities for Bax and Bcl-2 (MFIs), which were computed as the ratio of raw mean channel fluorescence to isotype control levels, respectively. The percentages of HeLa cells exhibiting the fluorescence were used to assess the cleaved caspase-3 concentrations in the treated and untreated cells. All results were presented as average values of three separate measurements [44].

#### 2.5.6. Statistical Analysis

The Shapiro–Wilk test was used to determine whether the distributions of the acquired data were normal. Mean ± standard deviation (SD) was used to present the MTT values and values of apoptotic proteins. The graph values of the Annexin-V/PI stained cells and values obtained by the cell cycle analysis were presented as means. For statistical analysis, commercial SPSS version 20.0 for Windows was utilized. The Student’s *t*-test for paired observations or a one-way ANOVA, depending on the data, was used for statistical evaluation. *p* values that were less than 0.05 were regarded as statistically significant.

## 3. Results and Discussion

### 3.1. General Procedure for the Synthesis of Thiourea Derivatives of Naproxen

**Scheme 1:**
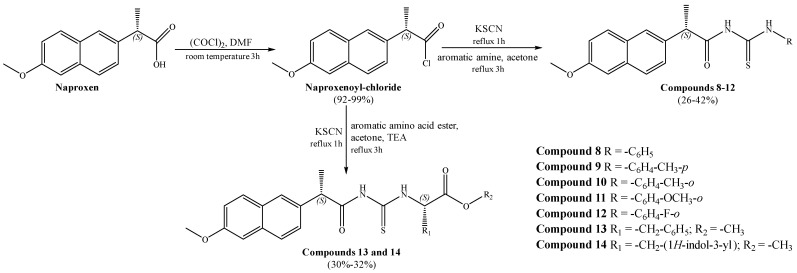
Synthetic procedure of compounds (**8**–**14**).

In the current study, seven novel thiourea derivatives (**8**–**14**) of naproxen were synthesized using a previously published synthetic procedure [37], as presented in Scheme 1.

The yields of compounds **8**–**14** were between 26 and 42%, which were very similar to the yields (25–43%) of previously synthesized compounds (**1**–**7**).

### 3.2. Acute Oral Toxicity Assessment

**Figure 1:**
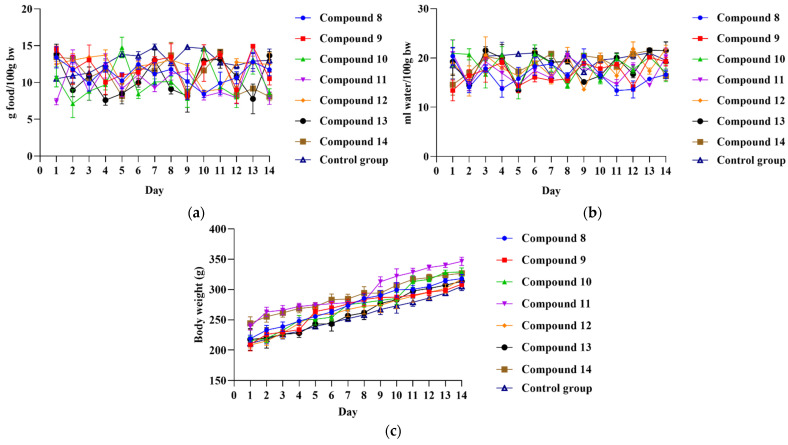
Results of food intake (**a**), water intake (**b**), and body weight (**c**) in treated and control rats.

During the 2-week observation period, no clinical signs of systemic toxicity, such as occurrence of aggressiveness, changes in urination, fur and skin, feces consistency, etc., were noticed in rats exposed to compounds **8**–**14**. Additionally, the intake of food and water, as well as weight gain, followed a similar trend in the control and experimental groups of rats. Moreover, relative organ weights between experimental and control rats did not significantly differ (Figure 1, Table 1).

### 3.3. In Vivo Assessment of the Anti-Inflammatory Potential of Novel Compounds ***8***–***14***

After accomplishing the acute oral toxicity study, the investigation of the anti-inflammatory potential of novel compounds **8**–**14**, which appeared to be safe at the applied dose of 10 mg/kg, was continued. The results of the anti-inflammatory potential for these compounds are shown in Table 2.

**Figure 2:**
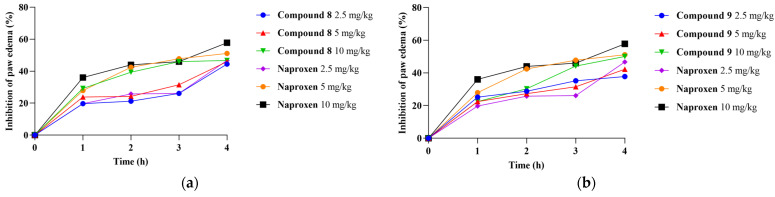

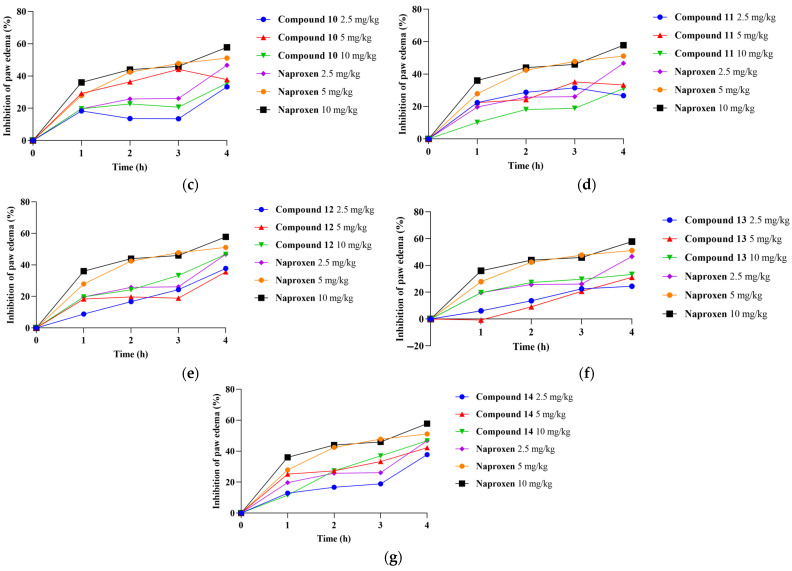
Percentage of paw edema inhibition in rats treated with 2.5 mg/kg, 5 mg/kg, and 10 mg/kg of compound **8** (**a**), compound **9** (**b**), compound **10** (**c**), compound **11** (**d**), compound **12** (**e**), compound **13** (**f**), compound **14** (**g**), and naproxen.

The obtained results revealed that paw edema, as an indicator of inflammation, was mostly affected by the dose, with the strongest decrease noticed in rats treated with the highest dose of all compounds. Moreover, the rise in paw edema was most intense during the first 2 h following carrageenan injection, where compounds **8**, **9**, **13**, and **14** in the highest dose exerted the greatest inhibitory potential compared to controls. Additionally, the prominent difference between untreated rats and rats that received novel compounds was observed after the 3rd and 4th hour following inflammation induction. In brief, compounds **8** and **9** were capable of decreasing paw edema, which was verified by a prominent percentage of inhibition, such as 44.83% and 49.29% respectively, at the end of the experiment. Understanding the mechanisms of acute inflammatory response is the basic step in proposing novel compounds as anti-inflammatory agents [45]. As relevant and widely used for testing novel compounds, carrageenan-induced paw edema represents an acute inflammation model characterized by biphasic edema. During the first hour, there is a release of histamine, serotonin, tachykinin, bradykinin, and other pro-inflammatory agents that immediately cause clinical manifestations such as edema, hyperalgesia, and erythema, appearing immediately [45,46]. This is in accordance with our observations, i.e., the greatest paw edema because of venous obstruction and elevated vascular permeability was noticed in all groups in the first two hours [47]. Nevertheless, in rats that received compounds **8** and **9**, paw edema was significantly alleviated (Figure 2).

On the other hand, prostaglandins production occurs later, and it has been proposed that carrageenan injection is responsible for TNF-α and IL-1 β generation, which stimulate prostaglandins synthesis by COX-2 [46]. The peak of the anti-edematous effects of novel compounds **8** and **9** in our research was revealed at the time of the most pronounced edema, in the 3rd and 4th hour following carrageenan injection. Oral application of these compounds appeared to be most efficient in reducing edema at the time when the edema is maximally developed. Therefore, we might assume that the considerable inhibition of paw edema, predominantly by **8** and **9**, during the delayed phase of inflammation (3rd and 4th hour) is a consequence of the interference of tested compounds with the biosynthesis of prostaglandins. However, future studies are necessary to provide a complete insight into the mechanisms of compounds **8** and **9** in the attenuation of inflammation response. Despite the fact that these two derivatives exhibited significant anti-inflammatory activity, their ability to reduce paw edema was still inferior compared to the most potent compound, the m-anisidine derivative (compound **4**), described in our previous publication [37]. Moreover, the anti-inflammatory activity of the tested compounds was still not comparable to the standard drug naproxen, which undoubtedly led to the highest percentage inhibition at all observed time points. Based on obtained results for both sets of compounds (**1**–**7** and **8**–**14**), it can be deduced that the presence of unsubstituted, m-substituted, and p-substituted aromatic amines in the side chain of investigated naproxen thiourea derivatives might be associated with potent anti-inflammatory activity.

### 3.4. Investigation of COX-2 and 5-LOX Enzyme Inhibitory Properties

The inhibitory potential of compounds **8**–**14** against COX-2 and 5-LOX enzymes was determined to assess the potential mechanism of their anti-inflammatory activity using fluorometric COX-2 and 5-LOX kits for the enzyme inhibition screening. Results of COX-2 and 5-LOX inhibition for compounds **8**–**14** are presented in Table 3 (IC_50_ values and percent of inhibition at 10 µM) as mean values of two measurements with standard errors.

The IC_50_ values could not be determined for any of the compounds **8**–**14**, indicating their weak COX-2 and 5-LOX inhibitory activity. However, if the percentages of COX-2 and 5-LOX inhibition for compounds **8**–**14** are compared, it can be concluded that they have a higher potential for 5-LOX inhibition. On the other hand, a previously published study showed that compounds **1**–**5** could be considered inhibitors of the 5-LOX enzyme according to their IC_50_ values that ranged from 0.30 µM to 39.77 µM, with derivative **4** achieving the strongest 5-LOX inhibition. Based on previous and current enzyme inhibition data, it can be concluded that synthesized compounds from both sets (**1**–**7** and **8**–**14**) did not exhibit significant COX-2 inhibition, while the most potent 5-LOX inhibitory activity was achieved by derivatives **1**–**5** [37].

### 3.5. In Vitro Cytotoxicity Studies

**Figure 3:**
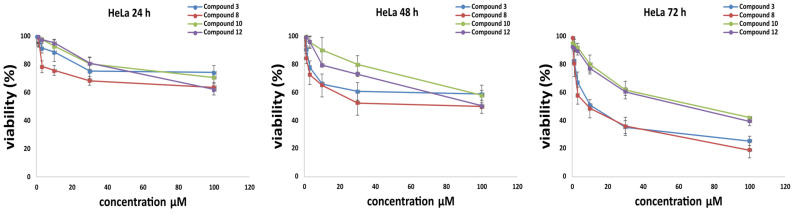
The cytotoxicity of compounds **3**, **8**, **10**, and **12** against HeLa cells after 24, 48, and 72 h. Data were expressed as mean ± standard deviation (mean ± SD).

The results of the current study showed that all fourteen tested compounds and naproxen displayed an insignificant effect on the viability of the MRC-5 fibroblast cell line (Table 4). In addition, most of the tested compounds showed a very weak cytotoxic effect against the MDA-MB-231 cell line, except compound **10**, which showed a moderate cytotoxic effect with IC_50_ values of 42 ± 9 µM, respectively. Compounds **3** and **8** significantly decreased the viability of the HeLa cell line after 72 h, with IC_50_ values of 11 ± 4 and 9 ± 2 respectively (Figure 3). Furthermore, compounds **10** and **12** showed a moderate cytotoxic effect against Hela cells. Compounds **2**, **4**, **9**, **10**, **12**, and **14** demonstrated moderate cytotoxic effects against the HCT 116 cell line. All other compounds included in the present research had an insignificant effect on the viability of the HCT 116 cell line. Based on the previously described results, it can be concluded that only two compounds, compounds **3** and **8,** showed strong cytotoxic effects only on the HeLa cell line (Figure 3). In line with that, it can be concluded that unsubstituted and p-substituted aromatic amines incorporated into the side chain of the thiourea moiety contribute significantly to cytotoxicity against the HeLa cell line.

**Figure 4:**
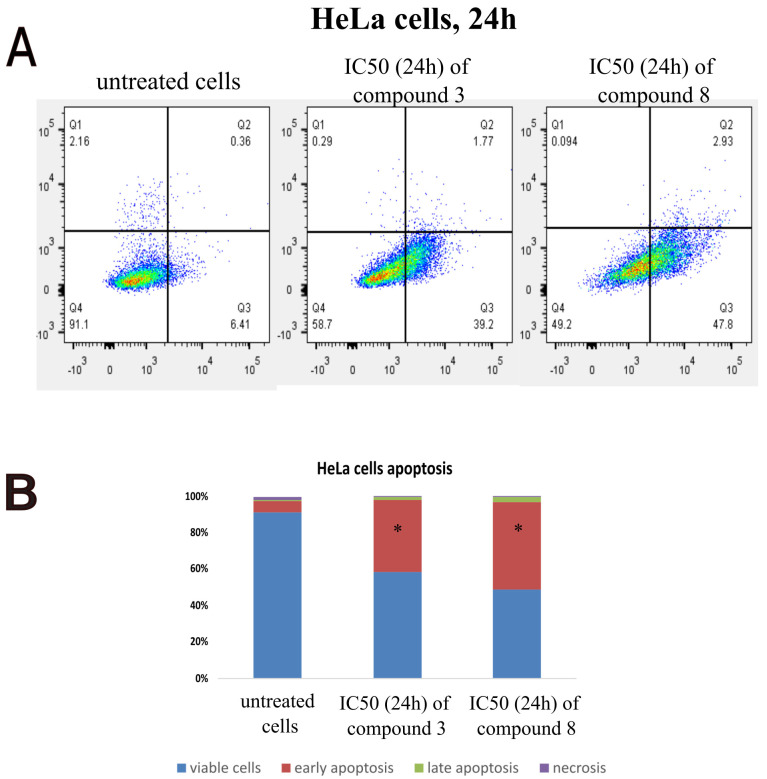
The flow cytometric analysis of Annexin V/PI stained HeLa cells. In untreated (control) and 24 h-treated (IC_50_ of compound **3** and **8**) HeLa cells, dot plots show the percentages of viable (lower left quadrant), early apoptotic (lower right quadrant), late apoptotic (upper right quadrant), and necrotic (upper left quadrant) cells. The findings are displayed as representative density plots of Annexin V-FITC and PI stainings (**A**) as well as in two-dimensional column charts (**B**) with average values of viable (blue), early apoptotic (red), late apoptotic (green), and necrotic cells (purple) from three separate experiments. * *p* < 0.05 compared to the untreated cells (control).

Therefore, the next step in the conducted study was to investigate the type of HeLa cell death induced by the two tested compounds. Results clearly showed that both compound **3** and compound **8** induced apoptosis after 24 h of treatment (*p* < 0.05, Figure 4). Furthermore, less than 1% of the HeLa cells were necrotic, and 1–3% of the tested cells were in late apoptosis (Figure 4).

**Figure 5:**
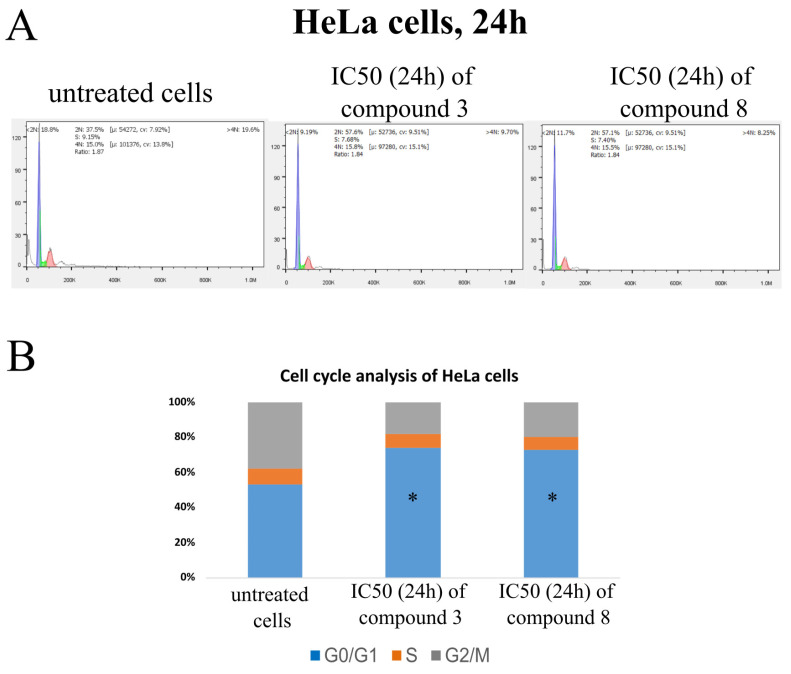
The effects of compounds **3** and **8** on cell cycle distribution of HeLa cells. In untreated (control) and 24 h-treated (IC_50_ of compound **3** and **8**) HeLa cells, the findings were shown as representative PI staining plots (**A**) and two-dimensional column charts (**B**) that show the average cell counts from three separate trials in the G0/G1 (blue), S (orange), and G2/M (grey) cell cycle phases. * *p* < 0.05 compared to the untreated cells (control).

The cell cycle plays an important role in cancerogenesis; thus, induction of cell cycle arrest may decrease the viability of tumor cells. In this study, cell cycle was explored 24 h after the treatment of HeLa cells with IC_50_ concentrations of two tested compounds by flow cytometry. Results demonstrated that both compound **3** and compound **8** induced G_0_/G_1_ phase arrest in HeLa cells (*p* < 0.05, Figure 5). We can conclude that the tested compounds decreased the viability of HeLa cells by inducing apoptosis and cell cycle arrest.

**Figure 6:**
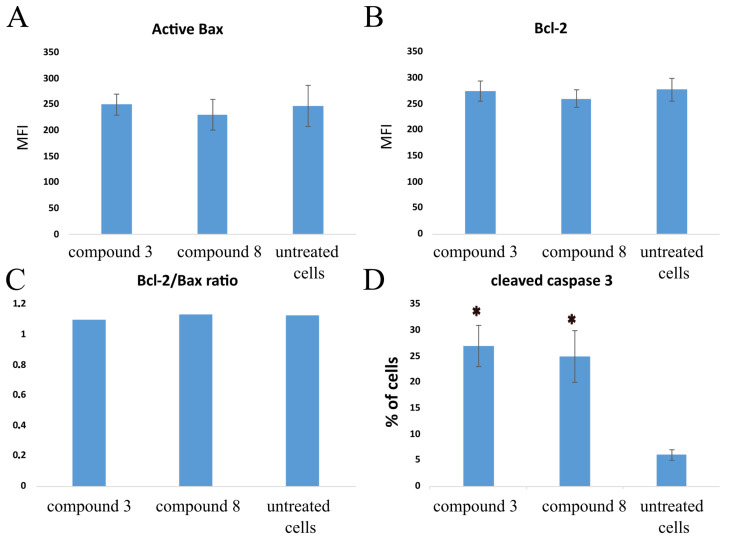
The effects of compounds **3** and **8** on the cellular concentration of proapoptotic protein active-bax, antiapoptotic protein bcl-2, and the percentage of HeLa cells displaying active caspase-3 after 24 h-treatment with IC_50_ values of respective compounds. (**A**) *MFI* values for pro-apoptotic protein active Bax of HeLa cells after 24 h-treatment with IC_50_ of compound **3** and compound **8**. (**B**) MFI values (mean fluorescence intensity) for anti-apoptotic protein Bcl-2 of HeLa cells treated with IC_50_ of compound **3** and compound **8**. (**C**) Bcl-2/Bax ratio for HeLa cells. (**D**) The percentages of cells displaying fluorescence for active (cleaved) caspase-3. The results were presented as mean ± SD of three separate experiments (**A**,**B**,**D**) or as medians (**C**). * *p* < 0.05 compared to the untreated cells (control).

Due to the fact that compounds **3** and **8** activate early apoptosis, the aim of this research was to determine whether tested compounds affected the cytoplasmic concentration of antiapoptotic protein Bcl-2 and proapoptotic protein Bax. Furthermore, active caspase 3 was evaluated in HeLa cells. The results after 24 h of treatment showed that all compounds insignificantly changed the amount of the antiapoptotic protein Bcl-2 and proapoptotic Bax (*p* > 0.05. Figure 6). Furthermore, the percentage of cells containing active caspase-3 was increased compared to the untreated cells (*p* < 0.05, Figure 6). Therefore, we can conclude that compounds **3** and **8** induce cell death through the involvement of the extrinsic pathway of apoptosis.

## 4. Conclusions

In this study, the synthesis of novel thiourea derivatives of naproxen with five aromatic amines and two aromatic amino acid esters (compounds **8**–**14**) was presented. The in vivo anti-inflammatory effect of the investigated compounds was predominantly dose-dependent, whereby the strongest decrease in paw edema was observed for the highest dose of all tested compounds. Results obtained from the in vivo assay indicated that derivatives **8** and **9** exhibited the most potent anti-inflammatory activity with a prominent percentage of inhibition, 44.83% and 49.29%, respectively. Moreover, the strongest inhibition of paw edema was observed during the delayed phase of inflammation, suggesting that the anti-inflammatory effect induced by investigated compounds is associated with the inhibition of prostaglandin biosynthesis. In vitro enzyme inhibition results demonstrated that neither of the newly synthesized compounds reached 50% inhibition of COX-2 and 5-LOX at concentrations lower than 100 μM. Considering the studies of cytotoxicity, all fourteen investigated compounds displayed an insignificant effect on the viability of the MRC-5 fibroblast cell and a very weak cytotoxic effect against the MDA-MB-231 cell line. On the other hand, derivatives **2**, **4**, **9**, **10**, **12**, and **14** demonstrated moderate cytotoxic effects against the HCT 116 cell line, while compounds **3** and **8** showed strong cytotoxic effects on the HeLa cells, suggesting involvement in the extrinsic pathway of apoptosis and G_0_/G_1_ phase arrest in HeLa cells. It can be concluded that the presence of unsubstituted, m-substituted, and p-substituted aromatic amines in the side chain of investigated thiourea derivatives of naproxen might be linked with potent anti-inflammatory and cytotoxic activity. According to results of biological activity in conducted studies, derivatives **4** and **8** are underlined as molecules with the strongest anti-inflammatory activity, while compounds **3** and **8** are the most promising cytotoxic agents. The present study provides valuable structural and biological data regarding investigated thiourea derivatives of naproxen and in that way, might speed up the design and discovery of new potent anti-inflammatory and cytotoxic agents.

## Figures and Tables

**Table 1:** Relative organ weights in treated and control rats.

Compound	8	9	10	11	12	13	14	Control Group
Organ								
Kidney	0.32 ± 0.02	0.29 ± 0.03	0.31 ± 0.04	0.33 ± 0.05	0.32 ± 0.04	0.28 ± 0.06	0.29 ± 0.04	0.35 ± 0.02
Heart	0.31 ± 0.01	0.29 ± 0.03	0.34 ±0.04	0.32 ± 0.02	0.35 ± 0.03	0.30 ± 0.04	0.31 ± 0.02	0.32 ± 0.01
Liver	2.65 ± 0.15	3.01 ± 0.27	2.67 ± 0.15	2.83 ± 0.20	2.76 ± 0.36	3.09 ± 0.27	2.03 ± 0.42	2.84 ± 0.09
Stomach	0.51 ± 0.06	0.56 ± 0.03	0.58 ± 0.04	0.54 ± 0.05	0.59 ± 0.05	0.50 ± 0.06	0.53 ± 0.04	0.55 ± 0.03

**Table 2:** Results of anti-inflammatory activity of compounds **8**–**14** in the carrageenan-induced rat paw edema model (mean value ± standard deviation (SD)).

Rat Paw Thickness (mm) (% of Inhibition)					
Experimental Groups	0 h	1 h	2 h	3 h	4 h
Compound **8** 2.5 mg/kg	3.70 ± 0.44	5.67 ± 0.15 (19.728%)	5.43 ± 0.15 (21.212%)	5.07 ± 0.21 (26.126%)	4.53 ± 0.50 (44.444%) *
Compound **8** 5.0 mg/kg	4.13 ± 0.31	6.0 ± 0.36 (23.810%) *	5.8 ± 0.44 (24.242%)	5.40 ± 0.44 (31.532%)	4.96 ± 0.35 (44.889%) *
Compound **8** 10.0 mg/kg	4.27 ± 0.15	6.0 ± 0.40 (29.252%) *	5.60 ± 0.44 (39.394%) *	5.27 ± 0.38 (45.946%) *	5.07 ± 0.21 (46.667%) *
Compound **9** 2.5 mg/kg	4.77 ± 0.15	6.60 ± 0.10 (25.170%) *	6.33 ± 0.06 (28.788%) *	5.97 ± 0.06 (35.135%)	5.70 ± 0.26 (37.778%)
Compound **9** 5.0 mg/kg	4.63 ± 0.29	6.53 ± 0.32 (22.449%)	6.23 ± 0.23 (27.273%)	5.90 ± 0.17 (31.532%)	5.50 ± 0.36 (42.222%) *
Compound **9** 10.0 mg/kg	4.30 ± 0.20	6.20 ± 0.17 (22.449%) *	5.83 ± 0.29 (30.303%) *	5.33 ± 0.15 (44.144%) *	5.05 ± 0.09 (50.000%) *
Compound **10** 2.5 mg/kg	4.17 ± 0.60	6.17 ± 0.51 (18.367%)	6.07 ± 0.83 (13.636%)	5.77 ± 0.85 (13.514%)	5.17 ± 0.75 (33.333%)
Compound **10** 5.0 mg/kg	4.73 ± 0.15	6.47 ± 0.51 (29.252%)	6.13 ± 0.47 (36.364%) *	5.77 ± 0.32 (44.144%) *	5.67 ± 0.40 (37.778%)
Compound **10** 10.0 mg/kg	4.23 ± 0.38	6.20 ± 0.36 (19.728%)	5.93 ± 0.31 (22.727%)	5.70 ± 0.44 (20.721%)	5.20 ± 0.44 (35.556%)
Compound **11** 2.5 mg/kg	5.40 ± 0.80	7.30 ± 0.60 (22.449%)	6.97 ± 0.67 (28.788%) *	6.67 ± 0.80 (31.532%)	6.50 ± 0.80 (26.667%)
Compound **11** 5.0 mg/kg	5.07 ± 0.32	6.97 ± 0.12 (22.449%)	6.73 ± 0.15 (24.242%)	6.27 ± 0.23 (35.135%)	6.07 ± 0.15 (33.333%)
Compound **11** 10.0 mg/kg	5.13 ± 0.32	7.33 ± 0.06 (10.204%)	6.93 ± 0.12 (18.182%)	6.63 ± 0.15 (18.919%)	6.17 ± 0.31 (31.111%)
Compound **12** 2.5 mg/kg	4.60 ± 0.36	6.83 ± 0.38 (8.844%)	6.43 ± 0.40 (16.667%)	6.00 ± 0.30 (24.324%)	5.53 ± 0.06 (37.778%)
Compound **12** 5.0 mg/kg	4.07 ± 0.06	6.07 ± 0.64 (18.367%)	5.83 ± 0.49 (19.697%)	5.57 ± 0.38 (18.919%)	5.03 ± 0.12 (35.556%)
Compound **12** 10.0 mg/kg	4.37 ± 0.38	6.33 ± 0.21 (19.728%)	6.03 ± 0.06 (24.242%)	5.60 ± 0.10 (33.333%)	5.17 ± 0.21 (46.667%) *
Compound **13** 2.5 mg/kg	4.70 ± 0.17	7.0 ± 0.10 (6.122%)	6.60 ± 0.10 (13.636%)	6.13 ± 0.12 (22.523%)	5.83 ± 0.06 (24.444%)
Compound **13** 5.0 mg/kg	4.43 ± 0.31	6.90 ± 0.10 (−0.680%)	6.43 ± 0.32 (9.091%)	5.90 ± 0.36 (20.721%)	5.47 ± 0.23 (31.111%)
Compound **13** 10.0 mg/kg	4.80 ± 0.36	6.77 ± 0.42 (19.728%)	6.40 ± 0.36 (27.273%) *	6.10 ± 0.36 (29.730%)	5.80 ± 0.30 (33.333%)
Compound **14** 2.5 mg/kg	4.40 ± 0.53	6.53 ± 0.42 (12.925%)	6.23 ± 0.25 (16.667%)	5.90 ± 0.26 (18.919%)	5.33 ± 0.49 (37.778%)
Compound **14** 5.0 mg/kg	4.93 ± 0.12	6.77 ± 0.38 (25.170%)	6.53 ± 0.42 (27.273%)	6.17 ± 0.25 (33.333%)	5.80 ± 0.10 (42.222%) *
Compound **14** 10.0 mg/kg	4.87 ± 0.25	7.03 ± 0.21 (11.565%)	6.47 ± 0.06 (27.273%) *	6.03 ± 0.25 (36.937%)	5.67 ± 0.21 (46.667%) *
1% DMSO	4.50 ± 0.09	6.95 ± 0.35	6.70 ± 0.32	6.35 ± 0.43	6.00 ± 0.32
Naproxen 2.5 mg/kg	4.38 ± 0.45	6.35 ± 0.15 (19.728%)	6.02 ± 0.15 (25.758%)	5.75 ± 0.20 (26.126%)	5.18 ± 0.20 (46.667%) *
Naproxen 5.0 mg/kg	4.25 ± 0.15	6.02 ± 0.20 (27.891%) *	5.52 ± 0.30 (42.424%) *	5.22 ± 0.30 (47.748%) *	4.98 ± 0.24 (51.111%) *
Naproxen 10.0 mg/kg	4.32 ± 0.32	5.88 ± 0.23 (36.054%) *	5.55 ± 0.45 (43.939%) *	5.35 ± 0.32 (45.946%) *	4.95 ± 0.35 (57.778%) *

* A statistically significant difference at the level of *p* < 0.05 in relation to the control group.

**Table 3:** Results of COX-2 and 5-LOX enzyme inhibition.

Compound	COX-2 Percent of Inhibition	5-LOX Percent of Inhibition
**8**	36.23 ± 16.45	34.63 ± 5.84
**9**	26.84 ± 6.07	12.94 ± 5.47
**10**	12.64 ± 4.99	27.92 ± 0.57
**11**	16.18 ± 13.72	31.24 ± 5.45
**12**	13.82 ± 6.41	29.72 ± 8.56
**13**	23.12 ± 9.83	19.84 ± 13.22
**14**	29.80 ± 12.12	46.24 ± 0.40

**Table 4:** IC_50_ values of compounds **1**–**14** and naproxen after 24, 48, and 72 h against MRC5, MDA-MB-231, HCT 116, and HeLa cells. Data were expressed as mean ± standard deviation (mean ± SD).

IC_50_ (µM)	MDA-MB-231			HeLa		
Compound	24 h	48 h	72 h	24 h	48 h	72 h
Compound **1**	>200	>200	>200	>200	>200	104 ± 8
Compound **2**	>200	>200	130.5 ± 10.4	>200	>200	>200
Compound **3**	>200	>200	>200	>200	120.5 ± 9.3	11.7 ± 4.3
Compound **4**	>200	>200	110.2 ± 12.9	>200	100.9 ± 12.2	90.8 ± 8.3
Compound **5**	>200	>200	120.3 ± 14.8	>200	160 ± 13	107 ± 10
Compound **6**	>200	>200	>200	>200	>200	>200
Compound **7**	>200	>200	>200	>200	>200	>200
Compound **8**	>200	>200	>200	>140.5 ± 7.2	97.6 ± 10.9	9.1 ± 2.5
Compound **9**	>200	>200	111.7 ± 18.1	>200	>200	120.4 ± 8.7
Compound **10**	100.9 ± 11.2	70.5 ± 8.3	42.2 ± 9.8	>200	110.7 ± 8.3	85.3 ± 9.1
Compound **11**	>200	>200	>200	>200	>200	>200
Compound **12**	>200	>200	>200	135.4 ± 16.8	103.2 ± 9.5	72.8 ± 6.9
Compound **13**	>200	>200	99.5 ± 8.2	>200	>200	>200
Compound **14**	>200	>200	117.4 ± 15.3	>200	150.7 ± 14.2	110.1 ± 11.5
Naproxen	>200	>200	>200	>200	>200	>200
**IC_50_ (µM)**	**MRC** **-5**			**HCT 116**		
**Compound**	**24 h**	**48 h**	**72 h**	**24 h**	**48 h**	**72 h**
Compound **1**	>200	>200	>200	>200	>200	>200
Compound **2**	>200	>200	>200	>200	110.8 ± 15.2	82.8 ± 10.6
Compound **3**	>200	>200	>200	>200	>200	>200
Compound **4**	>200	>200	>200	200	106.4 ± 12.9	71.6 ± 9.9
Compound **5**	>200	>200	>200	>200	157.7 ± 9.3	108.6 ± 14.2
Compound **6**	>200	>200	>200	>200	>200	>200
Compound **7**	>200	>200	>200	>200	>200	100.4 ± 11.3
Compound **8**	>200	>200	>200	>200	>200	100.7 ± 15.5
Compound **9**	>200	>200	>200	>200	105.2 ± 14.8	52.9 ± 8.1
Compound **10**	>200	>200	>200	120.4 ± 8.8	97.2 ± 6.8	33.3 ± 5.5
Compound **11**	>200	>200	>200	>200	>200	>200
Compound **12**	>200	>200	>200	>200	159.3 ± 17.5	68.8 ± 9.5
Compound **13**	>200	>200	>200	>200	>200	99.6 ± 4.9
Compound **14**	>200	>200	>200	150.2 ± 7.6	107.5 ± 12.7	47.1 ± 5.4
Naproxen	>200	>200	>200	>200	>200	>200

## Data Availability

Data are contained within the article.

## Supplementary Materials

The following supporting information can be downloaded at: https://www.mdpi.com/article/10.3390/pharmaceutics16010001/s1, Figure S1. ^1^H NMR spectrum (a) and ^13^C NMR (b) of compound 8 (MN19). Figure S2. ^1^H NMR spectrum (a) and ^13^C NMR (b) of compound 9 (MN21). Figure S3. ^1^H NMR spectrum (a) and ^13^C NMR (b) of compound 10 (MN22). Figure S4. ^1^H NMR spectrum (a) and ^13^C NMR (b) of compound 11 (MN23). Figure S5. ^1^H NMR spectrum (a) and ^13^C NMR (b) of compound 12 (MN27). Figure S6. ^1^H NMR spectrum (a) and ^13^C NMR (b) of compound 13 (MN28). Figure S7. ^1^H NMR spectrum (a) and ^13^C NMR (b) of compound 14 (MN25).
