# Supplementary material for: Synthesis, Characterization, and Investigation of Anti-Inflammatory and Cytotoxic Activities of Novel Thiourea Derivatives of Naproxen
Source: Pharmaceutics. 2023 Dec 19;16(1):1. doi: 10.3390/pharmaceutics16010001 (PMC10820527; doi:10.3390/pharmaceutics16010001)
Supplement: Supplementary file 1 [file pharmaceutics-16-00001-s001.zip › pharmaceutics-2735398-supplementary.pdf]

PHARMACEUTICS

Supplementary Information associated with the paper

Synthesis, Characterization, and Investigation of Anti-Inflammatory and Cytotoxic Activities of Novel Thiourea Derivatives of Naproxen

Nikola Nedeljković [1], Miloš Nikolić [1,*], Petar Čanović [2,*], Milan Zarić [2], Radica Živković Zarić [3], Jelena Bošković [4], Marina Vesović [1], Jovana Bradić [1,5], Marijana Anđić [1,5], Aleksandar Kočović [1,5], Marina Nikolić [5,6], Vladimir Jakovljević [5,6,7], Zorica Vujić [4], Vladimir Dobričić [4]

[1] Department of Pharmacy, Faculty of Medical Sciences, University of Kragujevac, Svetozara Markovica 69, 34000 Kragujevac, Serbia

[2] Department of Biochemistry, Faculty of Medical Sciences, University of Kragujevac, Svetozara Markovica 69, 34000 Kragujevac, Serbia

[3] Department of Pharmacology and Toxicology, Faculty of Medical Sciences, University of Kragujevac, Svetozara Markovića 69, 34000 Kragujevac, Serbia

[4] Department of Pharmaceutical Chemistry, Faculty of Pharmacy, University of Belgrade, Vojvode Stepe 450, 11221 Belgrade, Serbia

[5] Center of Excellence for Redox Balance Research in Cardiovascular and Metabolic Disorders, Svetozara Markovica 69, 34000 Kragujevac, Serbia

[6] Department of Physiology, Faculty of Medical Sciences, University of Kragujevac, Svetozara Markovica 69, 34000 Kragujevac, Serbia

[7] Department of Human Pathology, First Moscow State Medical University IM Sechenov, 119435 Moscow, Russia

[*] Correspondence: milos.nikolic@medf.kg.ac.rs (M.N.); petar.canovic@medf.kg.ac.rs (P.Č.)

Contents

1. NMR spectra of compounds 1-7.

^{1}H NMR spectrum (a) and ^{13}C NMR (b) of compound 8 (MN19). 3

^{1}H NMR spectrum (a) and ^{13}C NMR (b) of compound 9 (MN21). 4

^{1}H NMR spectrum (a) and ^{13}C NMR (b) of compound 10 (MN22). 5

^{1}H NMR spectrum (a) and ^{13}C NMR (b) of compound 11 (MN23). 6

^{1}H NMR spectrum (a) and ^{13}C NMR (b) of compound 12 (MN27). 7

^{1}H NMR spectrum (a) and ^{13}C NMR (b) of compound 13 (MN28). 8

^{1}H NMR spectrum (a) and ^{13}C NMR (b) of compound 14 (MN25). 9

a)

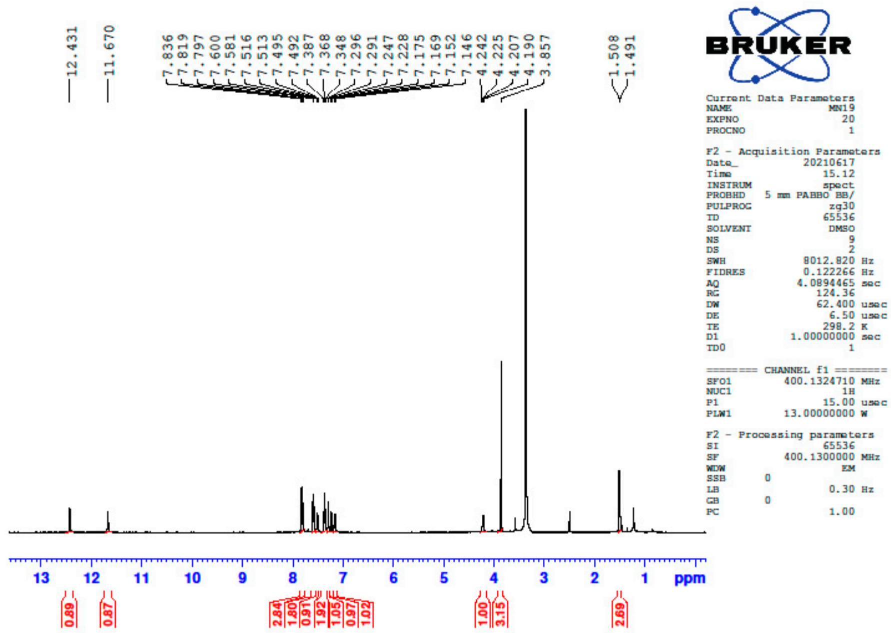

b)

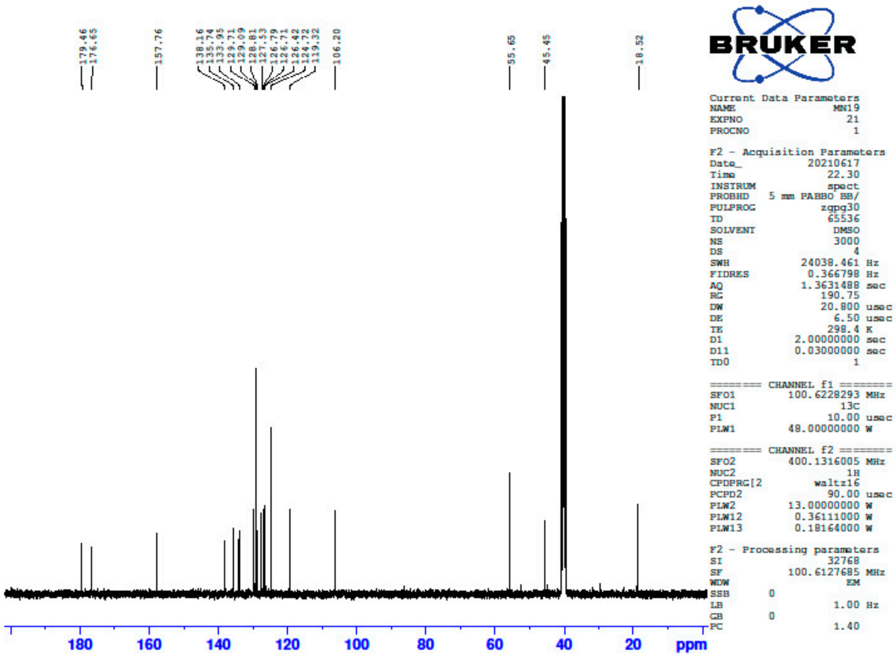

Figure S1. ^{1}H NMR spectrum (a) and ^{13}C NMR (b) of compound **8** (MN19).

a)

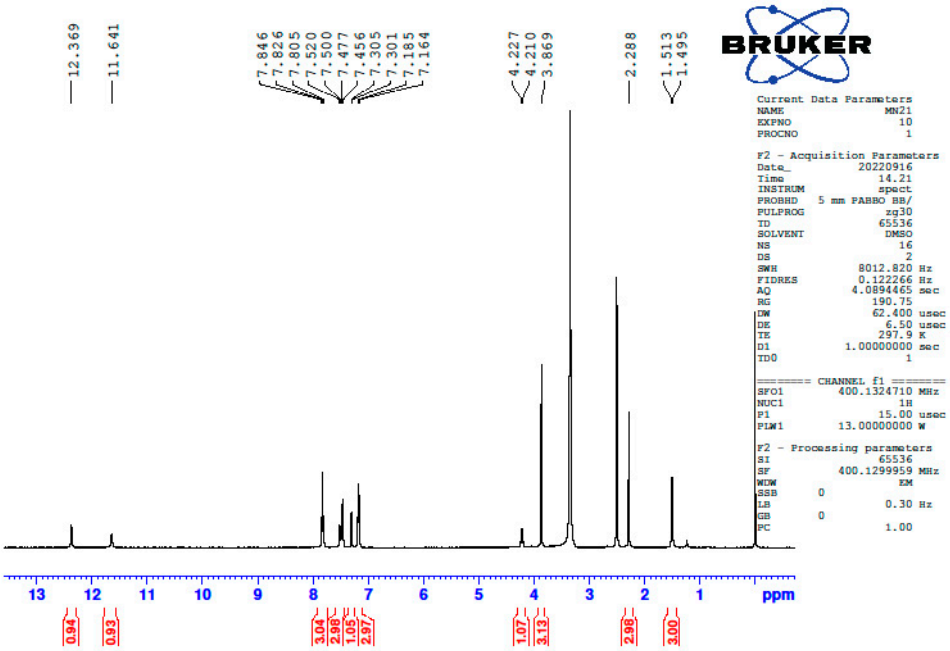

b)

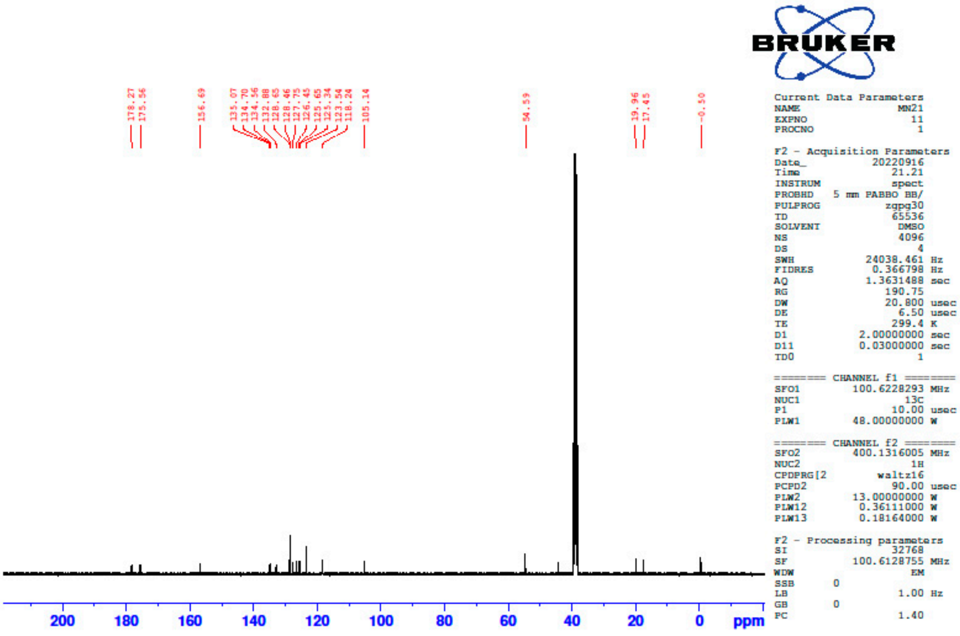

Figure S2. ^{1}H NMR spectrum (a) and ^{13}C NMR (b) of compound **9** (MN21).

a)

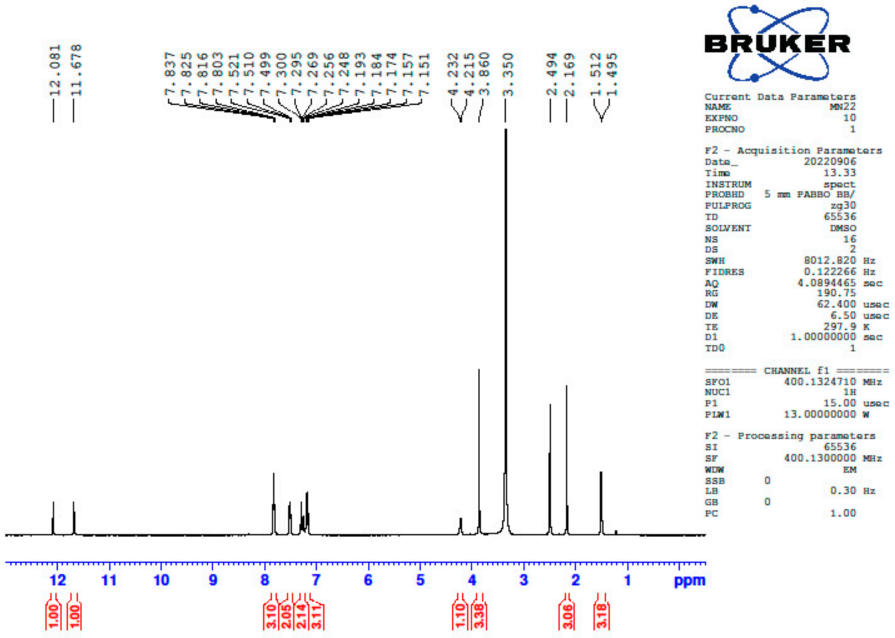

b)

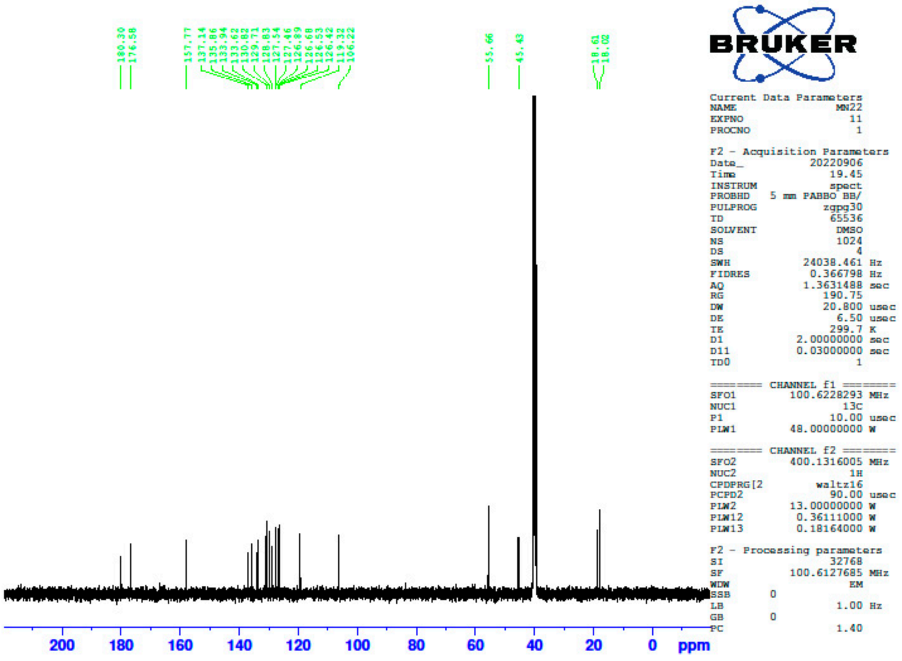

Figure S3. ^{1}H NMR spectrum (a) and ^{13}C NMR (b) of compound **10** (MN22).

a)

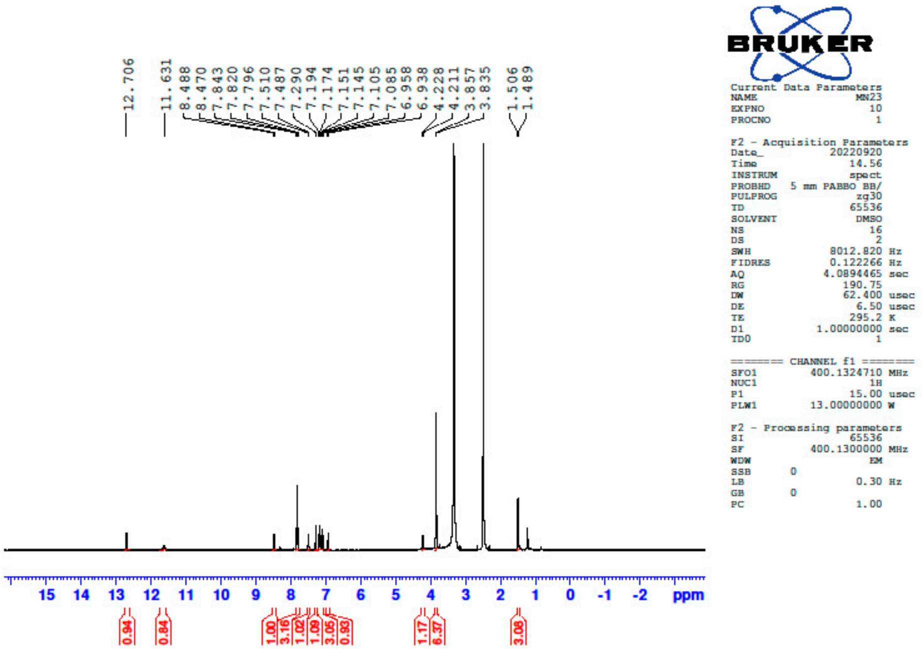

b)

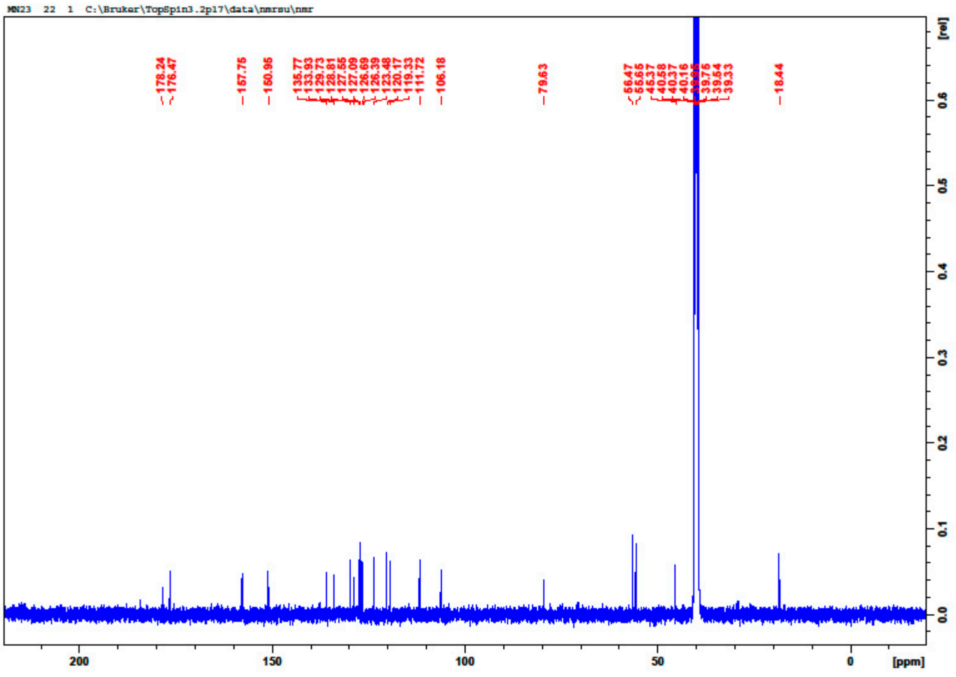

Figure S4. ^{1}H NMR spectrum (a) and ^{13}C NMR (b) of compound **11** (MN23).

a)

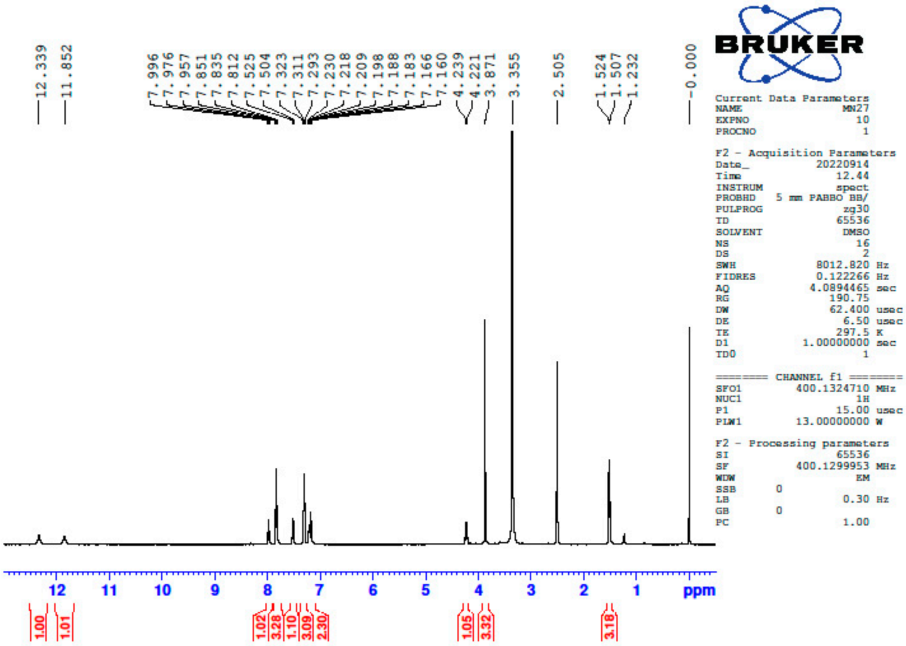

b)

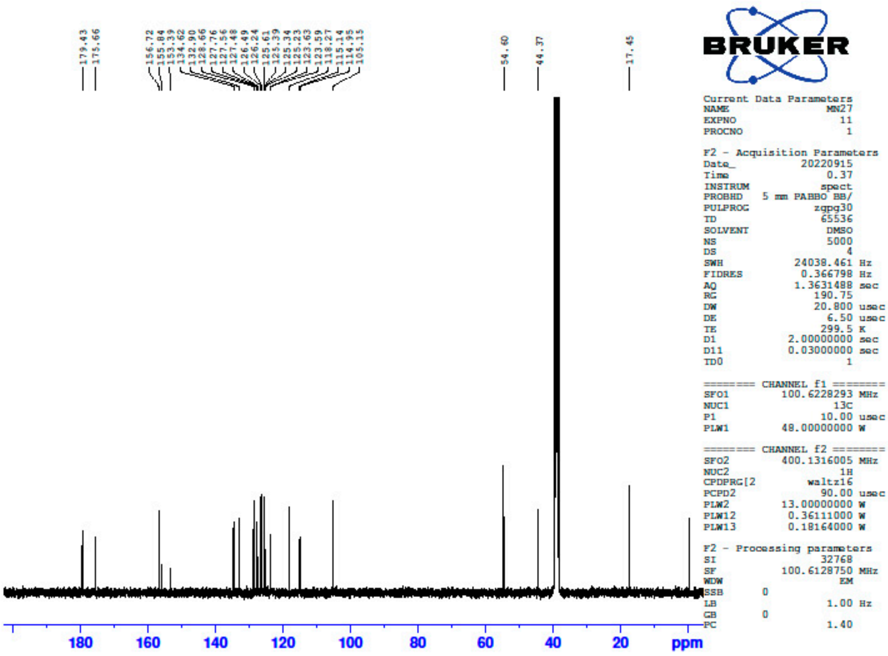

Figure S5. ^{1}H NMR spectrum (a) and ^{13}C NMR (b) of compound **12** (MN27).

a)

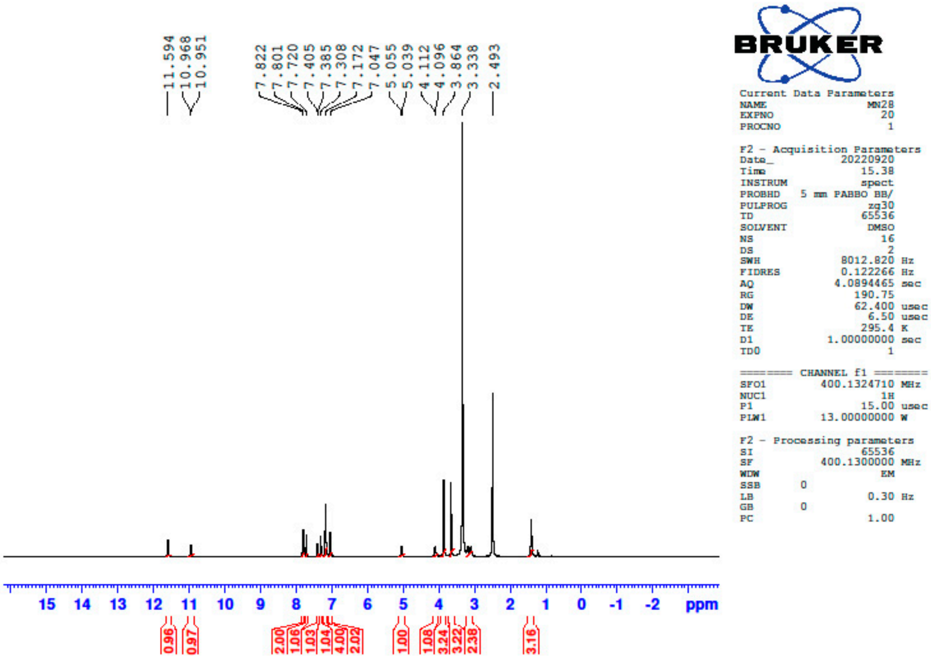

b)

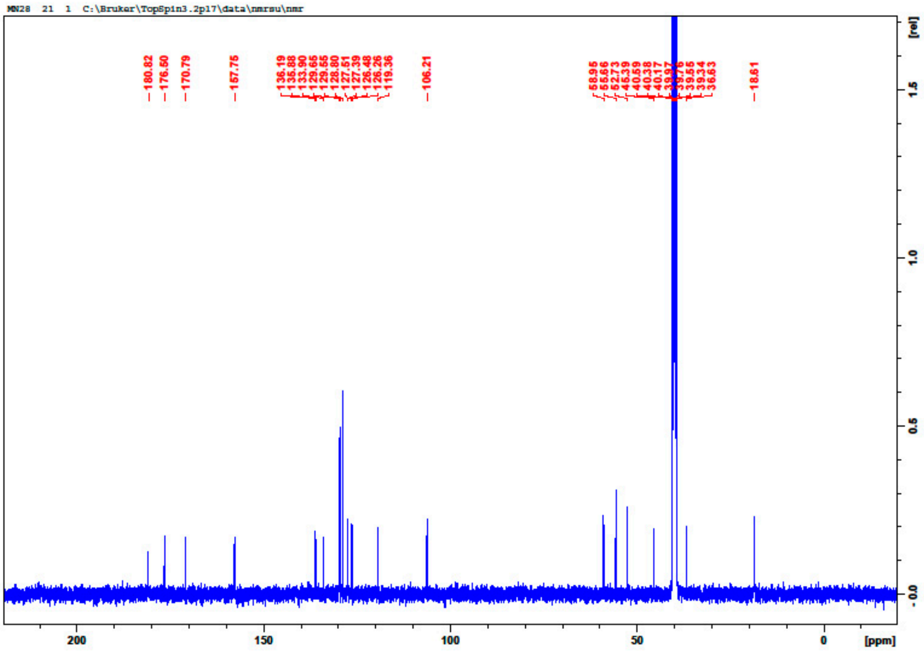

Figure S6. ^{1}H NMR spectrum (a) and ^{13}C NMR (b) of compound **13** (MN28).

a)

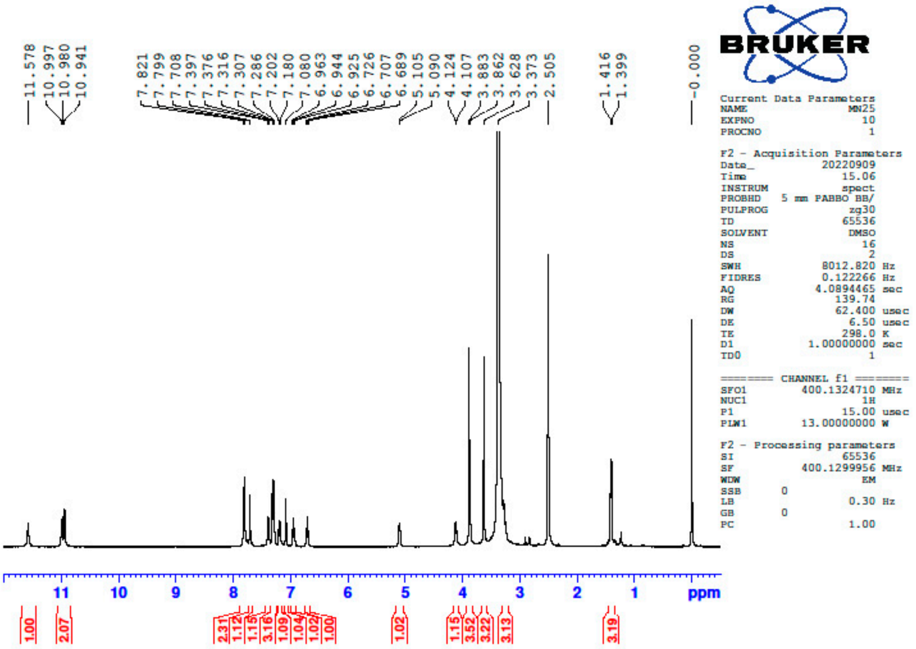

b)

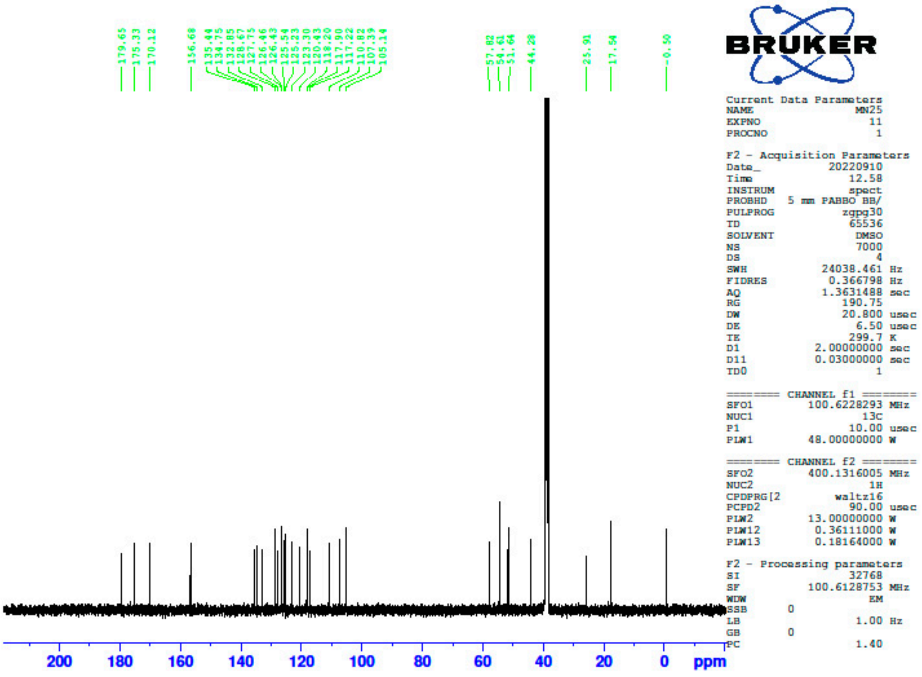

Figure S7. ^{1}H NMR spectrum (a) and ^{13}C NMR (b) of compound **14** (MN25).
